# THz-ATR Spectroscopy Integrated with Species Recognition Based on Multi-Classifier Voting for Automated Clinical Microbial Identification

**DOI:** 10.3390/bios12060378

**Published:** 2022-05-31

**Authors:** Wenjing Yu, Jia Shi, Guorong Huang, Jie Zhou, Xinyu Zhan, Zekang Guo, Huiyan Tian, Fengxin Xie, Xiang Yang, Weiling Fu

**Affiliations:** 1 Department of Laboratory Medicine, Southwest Hospital, Army Medical University (Third Military Medical University), Chongqing 400038, China; 18696791251@163.com (W.Y.); yollowrong@sina.com (G.H.); zhoujie950810@163.com (J.Z.); y13883402912@163.com (X.Z.); thylcq@163.com (H.T.); xiefengxin0310@163.com (F.X.); 2 Tianjin Key Laboratory of Optoelectronic Detection Technology and System, School of Electronic and Information Engineering, Tiangong University, Tianjin 300387, China; shijia@tiangong.edu.cn (J.S.); guozk805@126.com (Z.G.)

**Keywords:** terahertz spectroscopy, data analysis, microbial identification

## Abstract

The demand for rapid and accurate identification of microorganisms is growing due to considerable importance in all areas related to public health and safety. Here, we demonstrate a rapid and label-free strategy for the identification of microorganisms by integrating terahertz-attenuated total reflection (THz-ATR) spectroscopy with an automated recognition method based on multi-classifier voting. Our results show that 13 standard microbial strains can be classified into three different groups of microorganisms (Gram-positive bacteria, Gram-negative bacteria, and fungi) by THz-ATR spectroscopy. To detect clinical microbial strains with better differentiation that accounts for their greater sample heterogeneity, an automated recognition algorithm is proposed based on multi-classifier voting. It uses three types of machine learning classifiers to identify five different groups of clinical microbial strains. The results demonstrate that common microorganisms, once time-consuming to distinguish by traditional microbial identification methods, can be rapidly and accurately recognized using THz-ATR spectra in minutes. The proposed automatic recognition method is optimized by a spectroscopic feature selection algorithm designed to identify the optimal diagnostic indicator, and the combination of different machine learning classifiers with a voting scheme. The total diagnostic accuracy reaches 80.77% (as high as 99.6% for *Enterococcus faecalis*) for 1123 isolates from clinical samples of sputum, blood, urine, and feces. This strategy demonstrates that THz spectroscopy integrated with an automatic recognition method based on multi-classifier voting significantly improves the accuracy of spectral analysis, thereby presenting a new method for true label-free identification of clinical microorganisms with high efficiency.

## 1. Introduction

The rapid and accurate identification of pathogenic microorganisms is of particular importance for the prevention and treatment of important infectious diseases [1,2]. Currently, the gold standard for microorganism identification comprises cell culture methods, followed by biochemical assays designed to identify strains and species of microorganisms [3]. However, these biochemical assays are often limited by complicated processes, lengthy readout times, and the need for highly trained professionals [4]. In recent years, some rapid methods, including enzyme-linked immunosorbent assay (ELISA), polymerase chain reaction (PCR), and mass spectrometry (MS), have been applied in clinical microbial diagnosis [5]. Nevertheless, several unavoidable challenges exist, such as the need for costly reagents, cumbersome assay execution, and insufficiency of existing microbial databases [6]. Therefore, the development of a rapid and label-free identification strategy that does not require reagents or complex procedures would greatly improve the efficiency of clinical microbial diagnosis.

The development of modern optical technology has demonstrated the immense potential of a number of optical sensing systems for microbial identification. Among these, optical biosensors can sometimes be configured to yield rapid, label-free, multiplexed, and cost-effective diagnoses that are relatively free of experimental variability [1]. An emerging technology, terahertz (THz, 0.1–10 THz) spectroscopy, has the ability to probe intermolecular collective vibration modes (including vibrations defined by hydrogen bonds and van der Waals restoring forces) to evaluate the function and conformational characteristics of biomolecules in a label-free manner [7,8]. Given that the formation and breaking of the hydrogen bond network of water molecules occur on the picosecond scale, solvent water can generate strong THz absorption signals (~240 cm^−1^ at 1 THz) [9,10,11]. Consequently, THz spectroscopy has been extensively employed to investigate the dynamics of molecular hydration states, identification of tumor cells, differentiation of bacterial species, and delineation of dehydration processes of various biological tissues via progression through their different hydration states [12,13,14,15]. In particular, THz-attenuated total reflection (THz-ATR) spectroscopy acquires the THz signal of a sample, which is supported on an ATR prism using an evanescent wave that is concentrated within the range of tens of microns from the prism surface. The signal derives from THz radiation that is launched into the prism below its critical angle [16]. THz-ATR spectroscopy has been shown to be more sensitive than transmission or reflection THz spectroscopy when measuring highly absorptive biological samples [16,17]. Thus, THz-ATR spectroscopy has expected advantages (some of which have already been demonstrated in recent studies) in a number of different applications for the rapid and label-free detection of biological tissues, cells, and microorganisms [14,18,19]. For example, it has been used to determine the complex refractive indices of saccharide solutions and to experimentally characterize their global hydration states. The results indicate that the overall hydration state is closely related to the number of hydrophilic groups and to the steric configuration of hydroxyl groups in the saccharide units [20]. In addition to describing the hydration states of isolated biomolecules, the complex dielectric constants of cultured human cancer cells (DLD-1, HEK293, and HeLa) have been accurately determined by this method [21].

Our previous study demonstrated that fresh microbial samples lack distinct characteristic peaks, but present hydration-state-induced, distinguishable THz spectral profiles [22]. Based on this mechanism, a THz imaging study of single bacterial colonies showed that different bacterial species could be identified in a rapid and label-free manner [14]. However, the differences among the various hydration states alone could not be used for clinical microbial identification due to the strong heterogeneity seen in clinical strains that were isolated from different sample types and patient sources. Moreover, the presence of the spectral signatures of many mixed biological components in the samples complicates the THz spectra. This implies that the measured spectral signals need to be analyzed and interpreted to enable target identification. This effort requires developing an effective extraction and classification method with which to view the characteristic parameters of THz spectra to rapidly and accurately identify microbial species.

A previous study demonstrated that the use of principal component analysis (PCA) and random forest (RF) classifiers was helpful in the analysis of THz-ATR spectra for extracting features of human colorectal cancer cell lines [23]. The results indicate that the absorption coefficient is the most sensitive parameter for cancer cell discrimination [23]. In addition, the literature reveals that THz-ATR spectroscopy, integrated with PCA and quadratic discriminant analysis (QDA), can be used to identify DNA oligonucleotides with single-base mutations [24]. The reported work shows that various machine learning classifiers have diverse abilities for identifying different structures; however, THz-ATR spectroscopy integrated with an efficient classifier for clinical microbial recognition has not been reported to date.

Here, we propose an approach based on THz-ATR spectroscopic analytical technology integrated with an automated recognition method. Recognition is based on multi-classifier voting which we find to be useful for clinical microbial identification. The classifiers themselves each emphasize different inherent physical properties of the microorganisms. The THz-ATR spectra of five standard strains were obtained using a THz-ATR platform and automated recognition software. To improve the accuracy and efficiency of the recognition method, the diagnostic indicators of machine learning classifiers were optimized via feature selection based on the refractive index and absorption coefficient properties. Then, an automatic recognition operation based on multi-classifier voting was executed using these classifiers. THz-ATR spectra of the clinical samples of five common microorganisms (including 1123 isolates from clinical samples of sputum, blood, urine, and feces) were analyzed. The diagnostic performance of the automated recognition method with multi-classifier voting was compared with the results obtained using single classifiers. The results demonstrate that the proposed detection platform, combining THz-ATR spectroscopy and the automated recognition method based on multi-classifier voting, successfully identified five common clinical microorganisms, with a diagnostic accuracy of 80.77%.

## 2. Materials and Methods

### 2.1. Sample Preparation

Thirteen standard microbial strains, *Staphylococcus epidermidis* ATCC 12228, *Enterococcus faecalis* ATCC 29212, *Staphylococcus aureus* ATCC 25923, *Staphylococcus aureus* ATCC 29213, *Streptococcus pneumoniae* ATCC 49619, *Escherichia coli* ATCC 25922, *Pseudomonas aeruginosa* ATCC 27853, *Bacillus cereus* ATCC 11778, *Bacillus thuringiensis* ATCC 29730, *Bacillus subtilis* ATCC 6633, *Candida albicans* ATCC 10231, *Candida tropicalis* ATCC 13803, and *Candida glabrata* ATCC 15126, were purchased from the National Institute for the Control of Pharmaceutical and Biological Products (Beijing, China). A total of 1123 isolates of clinical microbial strains covering 5 species, including *E. coli*, *P. aeruginosa*, *C. albicans*, *C. tropicalis,* and *E. faecalis*, were isolated from clinical samples of sputum, blood, urine, and feces in the Clinical Microbiology Laboratory, Southwest Hospital of Army Medical University (Chongqing, China). A silicon (Si) container, the composition of which is the same as the Si prism, was fabricated as a sample cell for the THz-ATR spectrometer to measure a larger number of samples on a single prism and avoid the alignment errors caused by the need for Si prism replacement between samples. Resuscitated bacteria were inoculated on blood agar plates (CNA, Pang Tong, Chongqing, China) and cultured overnight at 37 °C. Bacterial colonies were then peeled from the plates and loaded into the Si container for measurement.

### 2.2. Set-Up of THz-ATR

**Figure 1:**
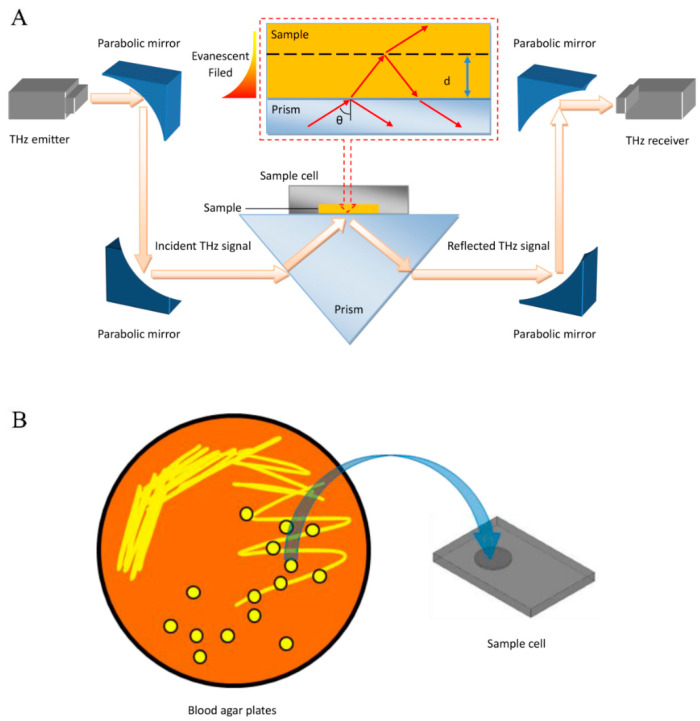
Identification of clinical microbes based on their THz-ATR spectra. (**A**) Schematic illustration of the THz-ATR spectrometer with a sample cell made of Si. (Inset) Diagram of the THz-ATR spectrometer with a “prism–sample” model. (**B**) Schematic showing sample loading.

The THz spectra of the bacterial samples were measured by a commercial THz time-domain spectroscopy (TDS) system (TAS 7500SP, Advantest Co., Tokyo, Japan) in ATR mode with a frequency range of 0.1–5.0 THz and a spectral resolution of 7.6 GHz, as shown in Figure 1A. Each sample was loaded into a Si container, which was fabricated as a circular sample well with a diameter of 7 mm and a depth of 100 μm to ensure uniformity of the sample thickness, as shown in Figure 1B. To carry out this measurement, the Si container was attached to the prism to enable the capture of information exclusively from the sample mass that was close to the prism surface. In contrast with transmission or reflection mode spectroscopy, the incident THz pulse is completely internally reflected off the surfaces of the ATR prism, and an evanescent field is created in the sample close to the sample–prism interface. Based on our previous studies, the penetration depth of the evanescent waves (approximately tens of micrometers around 0.1–5.0 THz) is much smaller than the thickness of the examined sample (approximately 100 μm); thus, we can use the “prism–sample” (inset) model to obtain the THz absorption coefficient of the bacterial samples [25,26]. Fresnel’s reflection coefficient (${\tilde{r}}_{12}$) of the prism–bacteria interface can be obtained by calculating the reflectance ($\tilde{R}$) and phase spectrum ($\tilde{\varphi }$) of the THz time-domain spectrum after Fourier transform, as shown in Equations (1) and (2):

(1) $$\tilde{R}={\left|\frac{{\;\tilde{r}}_{12}}{r_{REF}}\right|}^{2}$$

(2) $$\tilde{\varphi }=Arg\left[\frac{{\;\tilde{r}}_{12}}{r_{REF}}\right]$$

where $r_{REF}$ is the reflection coefficient of the prism–air interface (reference signal). ${\tilde{r}}_{12}$ can also be calculated as a function of the incident angle (θ) of the THz wave, and of the (complex) refractive index of the ATR prism ($\varepsilon_{1}$) and sample (${\tilde{\varepsilon }}_{2}$). The complex permittivity of the examined sample is obtained after determination of these parameters, as shown in Equation (3):

(3) $${\tilde{r}}_{12}=\frac{\sqrt{\varepsilon_{1}}\;\sqrt{1-\left(\frac{\varepsilon_{1}}{{\;\tilde{\varepsilon }}_{2}}\right)\sin^{2}\theta \;}-\sqrt{{\;\tilde{\varepsilon }}_{2}}\cos\theta }{\sqrt{\varepsilon_{1}}\;\sqrt{1-\left(\frac{\varepsilon_{1}}{{\;\tilde{\varepsilon }}_{2}}\right)\sin^{2}\theta \;}+\sqrt{{\;\tilde{\varepsilon }}_{2}}\cos\theta }$$

The relationship between the extinction coefficient (κ) and refractive index (n) of the examined sample and the imaginary part (ε″) and real part (ε′) of the complex permittivity ($\tilde{\varepsilon }$) of the examined sample can be obtained as shown in Equations (4) and (5):

(4) $$\;\varepsilon \prime =n^{2}-\kappa^{2}$$

(5) ε″=2nκ

Additionally, the absorption coefficient (α) of the examined sample can be deduced from the extinction coefficient, the angular frequency (ω), and the speed of light (c), as shown in Equation (6):

(6) $$\alpha =\frac{2\omega \kappa }{c}$$

In this case, the THz-ATR spectrum originating from the bare sample cell was taken as the reference signal, and the THz-ATR spectrum of the loaded sample produced the measured signal. The container was rinsed with absolute ethanol and ultrapure water and dried with N2 after each measurement. To increase reliability, each sample was measured six times, and then the averaged spectra for every sample were used for further analysis. The experiments were performed at room temperature (26 ± 0.5 °C) and an appropriate humidity (<5%). The total test time after cell culture was less than 3 min, including 1 min for sample loading, 1 min for spectral acquisition, and 1 s for data analysis.

### 2.3. Statistical Analysis

The automatic recognition procedure was performed by multi-classifier voting based on three types of classifiers: a *k*-nearest neighbor (*k*NN) classifier, a support vector machine (SVM) classifier, and a random forest (RF) classifier. The *k*NN algorithm is a nonparametric classification method based on a calibration data set [27]. In *k*NN classification, the result of THz-ATR spectroscopy is classified as belonging to the majority class of the *k* nearest neighbors in the feature space. This method is appropriate for pattern recognition with a large sample size. The SVM algorithm is a machine learning approach based on the structural risk minimization principle [28]. An SVM classifier is realized by mapping the spectral feature results to a high-dimensional space to facilitate separation in the feature space based on the maximum interval hyperplane. This method is appropriate for nonlinear and high-dimensional feature pattern recognition with a small sample size. RF is an integrated learning method for classification and regression that constructs many decision trees and outputs the pattern of classes (classification) or average prediction (regression) of the individual trees [29]. This method is appropriate for large sample sizes and unmarked pattern recognition features [30].

The results of the classification descriptions for the proposed automatic recognition method were analyzed by evaluating the accuracy, receiver operating characteristic curve (ROC), and the area under the curve (AUC) that each working classification produced. The ROC curve and AUC scores were used to evaluate the capability of the recognition method to generalize the empirical data. The ROC curve is defined as the true positive rate (TPR) against a given false positive rate (FPR). The AUC score is defined as the area under the ROC curve.

## 3. Results and Discussion

### 3.1. THz-ATR Absorption Spectra of Standard Strains

**Figure 2:**
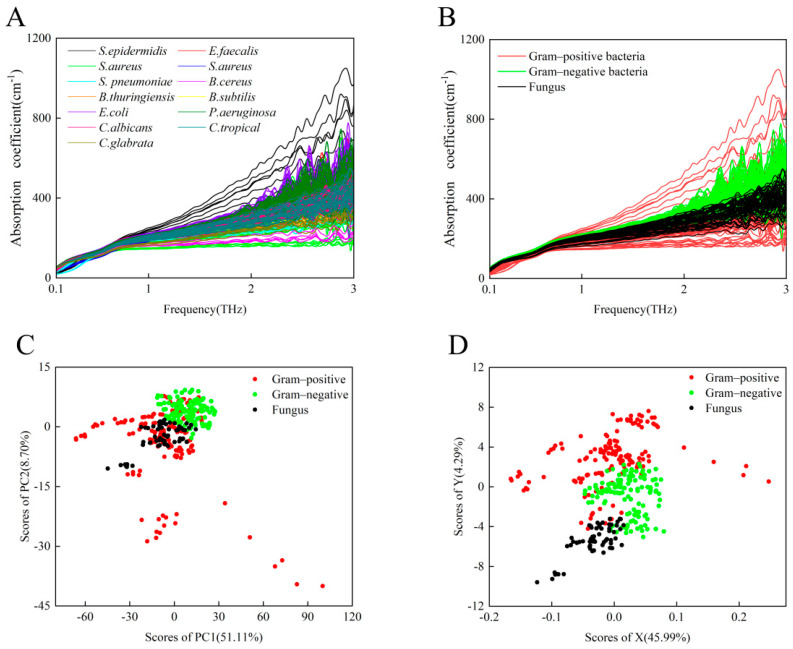
(**A**) A total of 387 THz absorption spectra curves of 13 species of standard microbial strains. (**B**) THz absorption spectra of eight Gram-positive bacterial strains (red), two Gram-negative bacterial strains (green), and three fungi (black). (**C**) PCA of THz spectra for Gram-positive bacterial strains (red), Gram-negative bacterial strains (green), and fungi (black). (**D**) Least-squares analysis representation of THz spectra for Gram-positive bacterial strains (red), Gram-negative bacterial strains (green), and fungi (black).

Figure 2A shows that 387 THz absorption spectra curves of 13 species of standard strains exhibit overlapping spectra that increase monotonically with frequency, especially in the lower-frequency band from 0.1 to 1.0 THz. As shown in Figure 2B, there was persistently no significant difference in the THz-ATR absorption spectra among the groups after the 13 standard bacteria were classified. The category classes were: Gram-positive bacteria (including *S. epidermidis*, *E. faecalis*, *S. aureus*, *S. pneumoniae*, *B. cereus*, *B. thuringiensis*, and *B. subtilis*), Gram-negative bacteria (including *E. coli* and *P. aeruginosa*), and fungi (including *C. albicans*, *C. tropicalis*, and *C. glabrata*). As expected, although a few bacterial species can be identified by the variation in hydration state that was associated with the THz absorption coefficients, it is difficult to accurately identify microorganisms solely by their THz absorption due to the possibility of potentially similar hydration states as the number of sampled species increases [23].

We used the common PCA method and the least-squares analysis method to resolve the THz spectra and distinguish these three groups of microorganisms. PCA is a common unsupervised classification method that is often used for spectral data analysis to reduce the dimension or number of variables in a multi-dimensional data set [31]. The least-squares analysis method is a supervised learning method that analyzes the different relationships between one dependent variable and several independent variables; it is often applied to characterize relationships in bioinformatics and chemistry [32]. The PCA results are shown in Figure 2C. Significant differences were observed between the Gram-negative bacteria and fungi; however, the distribution of Gram-positive bacteria overlaps with those of the other two groups. Although a small number of samples overlap, the three different groups of microorganisms can be preliminarily differentiated by the least-squares method, as shown in Figure 2D. This suggests that other data processing methods might contribute to differentiating similar hydration-state-induced THz absorption spectra of multiple microbial species.

### 3.2. THz-ATR Absorption Spectra of Clinical Strains

**Figure 3:**
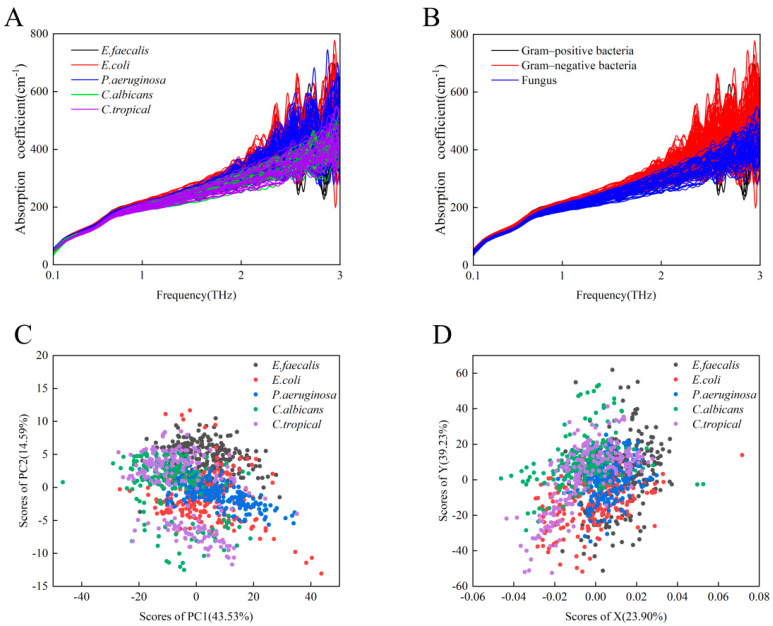
(**A**) A total of 1123 THz absorption spectra curves of 5 species of clinical strains, specifically *E. faecalis* (black), *E. coli* (red), *P. aeruginosa* (blue), *C. albicans* (green), and *C. tropicalis* (purple). (**B**) THz absorption spectra of one Gram-positive bacterial strain (black), two Gram-negative bacterial strains (red), and two fungi (blue). (**C**) PCA representation of the THz spectra for the above five clinical strains. (**D**) Least-squares analysis representation of the THz spectra for the above five clinical strains.

Taking into account the diversity of patient sources and the likelihood that the genotypes and phenotypes of the same microbe from different sample sources may be slightly different, clinical microbial strains should exhibit greater general heterogeneity than standard microbial strains. To investigate the potential of THz-ATR spectroscopy for the diagnosis of clinical strains, we recorded the THz-ATR absorption spectra of 1123 clinical strains belonging to the aforementioned three classes of bacteria and two classes of fungi. As shown in Figure 3A, it is difficult to distinguish these five clinical microbial strains based on their absorption coefficients alone. As shown in Figure 3B, the overall THz absorption spectra of the two species of Gram-negative bacteria (*E. coli* and *P. aeruginosa*) appear to be slightly more distinguishable than the spectra of some fungi (*C. albicans* and *C. tropicalis*), whereas the set of absorption coefficients of the Gram-positive bacterium (*E. faecalis*) overlaps with those of the other two groups. We further analyzed the THz absorption of five species of clinical strains by the PCA and least-squares methods, as shown in Figure 3C,D. These results show that neither PCA nor the least-squares method effectively distinguishes the five clinical strains, suggesting that it is necessary to apply another learning algorithm to analyze the sets of THz spectra from multiple clinical strains.

### 3.3. Automated Recognition of Clinical Strains

**Figure 4:**
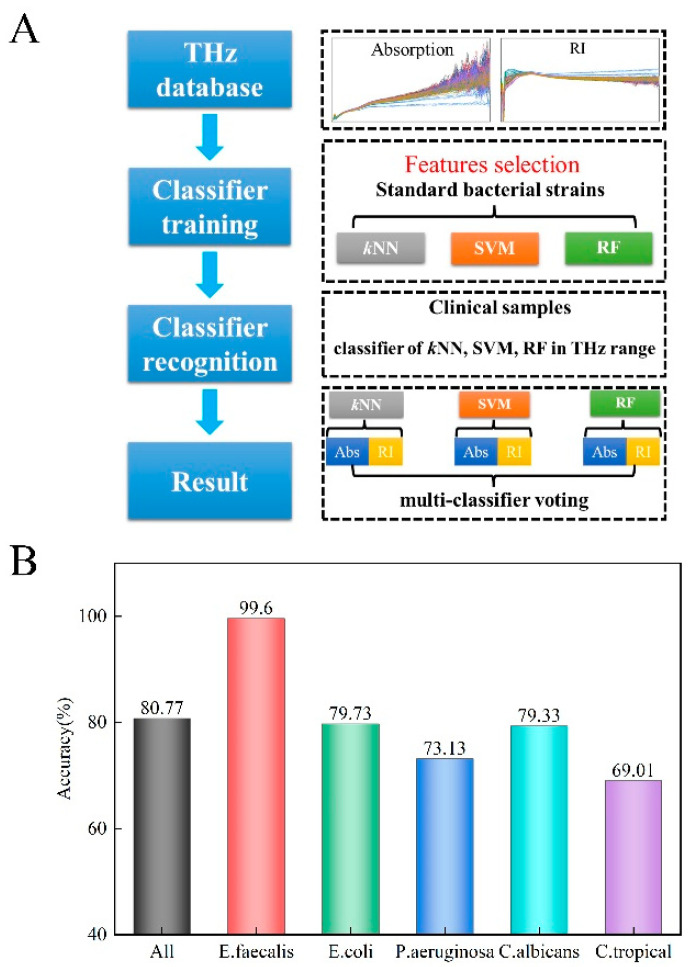
(**A**) The proposed automated recognition workflow for constructing the THz database of common microorganisms. (**B**) Discrimination function filter of the automatic recognition method is based on the multi-classifier voting scheme for clinical strain diagnosis.

An automated recognition method, based on multi-classifier voting, was developed using the classifiers *k*NN, SVM, and RF, as shown in Figure 4A. THz-ATR spectra were used as the diagnostic indicator of the classifiers. Spectroscopic features (frequency range of 0.1–5.0 THz) of the five standard strains, including the refractive index and absorption coefficient, were chosen to comprise the training set, respectively, and used to construct the automated recognition algorithm. Then, six groups of results were used in the voting of the multi-classifier predictive model. To improve predictive accuracy and efficiency, an algorithm was written that executes spectroscopic feature selection for the diagnostic indicator of each classifier. Here, the ReliefF algorithm is used in the feature selection for dimensionality reduction instead of PCA, which is preferred in supervised identification applications. Spectroscopic features for the refractive index and absorption coefficients were ranked by a weighing factor that was calculated by the ReliefF algorithm, where a specific refractive index and absorption coefficients were selected according to the criterion of achieving minimal error until maximal achievable accuracy was obtained [33]. That is, the two data sets for each classifier were ranked by the weighting factor, and different elements from each data set were chosen as diagnostic indicators of each classifier grouping according to the particular value of the corresponding weighing factor that achieves the highest diagnostic accuracy [33]. The ultimate result was obtained by voting of the results from these six groups of results.

The measured THz-ATR spectra of the 1123 clinical samples (including 253 for *E. faecalis*, 222 for *E. coli*, 227 for *P. aeruginosa*, 208 for *C. albicans,* and 213 for *C. tropicalis*, Table 1) were used as a validation set to evaluate the discrimination performance of the automated method. The results of traditional microbial culture identification methods were employed as the gold standard basis set.

Figure 4B shows the overall diagnostic accuracy when the above procedure was employed. We found that the average accuracy was 80.77% across the five clinical strains (99.60% for *E. faecalis*, 79.73% for *E. coli*, 73.13% for *P. aeruginosa*, 79.33% for *C. albicans,* and 69.01% for *C. tropicalis*). Park et al. analyzed some results of recently developed machine learning (ML)-based THz applications, and the current ML techniques were approximately 90% sensitive for diagnosing a disease according to their review [34]. Peiffer-Smadja et al. explored 97 ML systems aiming to assist clinical microbiologists, among which, only one ML system was reported to be used in clinical practice [35]. This ML system aimed to screen urine samples, the analysis results from 212,554 urine reports showed the potential for using machine learning algorithms, and the classification sensitivity was >95% [36]. The average accuracy of our automatic identification method is no higher than that in the aforementioned reports. We hypothesize that this is related to the fact that clinical strains have greater sample heterogeneity than standard strains. The measured samples were isolated from clinical samples of different sources, including sputum, blood, urine, and feces in our laboratory, the genotype and phenotype of the same microbe from different sample sources may be slightly different.

### 3.4. Optimizing the Classification Scheme of the Automated Recognition Method

**Figure 5:**
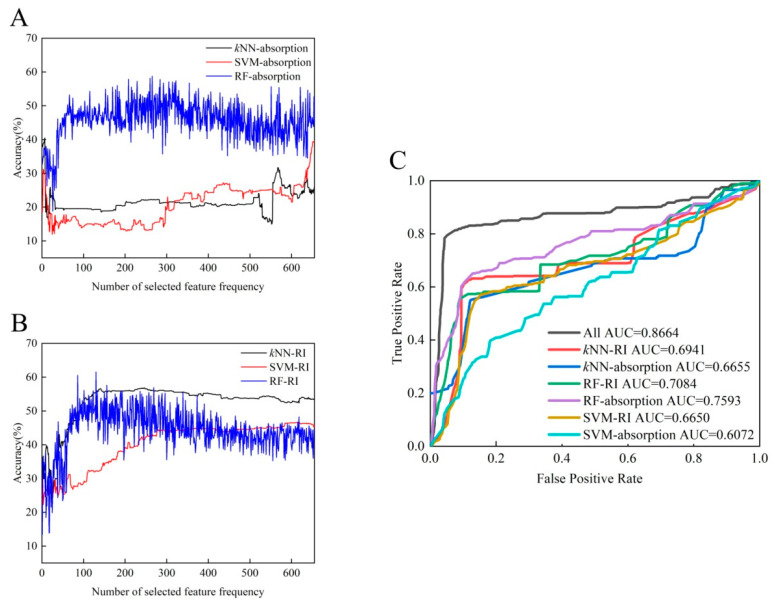
Diagnostic evaluation of the automated recognition method based on two characteristic parameters: absorption (**A**) and refractive index (**B**). (**C**) ROC curves and AUC scores for identifying clinical strains with three types of machine learning classifiers.

Based on an inspection of the microbial identification results, we tried to reduce the dimensionality of the THz-ATR data set using different parameters that characterize the spectral features (including the absorption coefficient and refractive index) and to subsequently probe the accuracy of the diagnosis using each of the classifiers. The corresponding accuracies for species recognition, based on the *k*NN, SVM, and RF classifiers, are shown in Figure 5A,B, as a function of the number of selected feature frequencies. As shown in Table 2, the diagnostic accuracy of the classifier with the refractive index as the characteristic feature indicator is greater than that of the absorption coefficient feature indicator. This inspired us to consider additional indicators as comprehensive parameters of the THz-ATR spectrum that might improve the diagnostic accuracy. We turned our attention to additional parameters such as the dielectric loss, the imaginary and real parts of the complex permittivity, and the power, which could conceivably lead to the discovery of diagnostic indicators for the corresponding classifier [22]. To achieve the maximum contribution to the multi-classifier algorithm and the highest diagnostic accuracy, each classifier algorithm selected the optimal number of extracted characteristics (details in Table 2). The ROC diagnostic curve plots the variation in the true positive vs. false positive rates using each of the machine learning classifiers, as shown in Figure 5C. Among these, the RF classifiers achieved the highest accuracy with an appropriate use criteria (AUC) score of 0.7593. However, the total classification accuracy of the proposed automated recognition method was found to be significantly better when based on the combined multi-classifier voting scheme than on a single classifier scheme (AUC 0.8664 vs. 0.7593). The use of all classifiers rather than a single classifier reduced the misdiagnosis and missed diagnosis rates for identifying clinical samples as determined by higher AUC scores. Therefore, we conclude that automated identification of these five clinical strains from their THz-ATR spectra can be significantly improved by establishing a multi-classifier voting scheme and screening for the best characteristic parameters that highlight differences in the sample populations. Our future work will also use other classifiers (such as artificial neural networks, Bayesian learning, and decision trees), to test whether they contribute positively or negatively to improving accuracy in identifying clinical microorganisms.

## 4. Conclusions

In this study, we presented a novel strategy, based on THz-ATR spectroscopy that is integrated with an algorithm for automated data recognition to identify microbiological species of clinical importance. Our results demonstrate that pathogenic microorganisms can be characterized by their THz-ATR spectra in a label-free manner. When combined with the least-squares method for analyzing spectral features, 13 standard strains can be divided into three different groups: Gram positive bacteria, Gram negative bacteria, and fungi. However, considering that clinical microbial strains generally have greater heterogeneity than standard microbial strains, their THz-ATR absorption spectra are not differentiated by common PCA and the least-squares analysis methods alone. We therefore developed an automatic recognition method based on multi-classifier voting to analyze THz spectra based on several intrinsic physical properties. Using traditional culture and biochemical assays as the gold standard, the feasibility of this method for automated clinical microbial strain identification was systematically evaluated. The diagnostic accuracy and validity for 1123 different specimens were found to exceed 80% when using a minimum of three different classifier representations. The routine clinical method of bacterial identification is based on biochemical and metabolic profiling, which requires 24–48 h [37]. As spectral acquisition and data analysis can be completed in 1 min after bacterial culture is complete, our proposed strategy is advancing at least 10 h compared to the traditional culture-based methods, because it reduces the time consumption of biochemical assays. MS in clinical laboratories is also a culture-based method. Microbial identification by MS is performed by database matching; however, the turnaround time for the identification of bacterial isolates from colonies ranges between 5 and 45 min, depending on whether a protein extraction step is required [38]. Compared with MS, our strategy reduces the sample preparation time and simplifies the workflow. In addition, our assay utilizes the physical properties of microbial cells, and the target bacterium can be identified without any need for pretreatment or reagents; thus, compatibility with downstream analytical techniques can be ensured. With the continuous improvement and supplementation of the spectral database and classifiers, this method has potential for many applications in the early clinical survey and diagnosis of pathogenic and non-pathogenic microbes.

## Figures and Tables

**Table 1:** Classification results obtained by the multi-classifier voting scheme.

	*E. faecalis*	*E. coli*	*P. aeruginosa*	*C. albicans*	*C. tropicalis*	Total
Standard strains for modeling	25	29	27	26	30	137
Clinical strains for identification	253	222	227	208	213	1123
Correctly identified strains	252	177	166	165	147	907

*E. faecalis*: *Enterococcus faecalis*; *E. coli*: *Escherichia coli*; *P. aeruginosa*: *Pseudomonas aeruginosa*; *C. albicans*: *Candida albicans*; *C. tropicalis*: *Candida tropicalis*.

**Table 2:** Classification accuracy of the three types of classifiers for the five clinical strains.

Classifier	Parameter	Accuracy (%)	Number of Extracted Characteristics
*k*NN	RI	56.1%	266
*k*NN	Absorption	40.5%	7
SVM	RI	46.6%	610
SVM	Absorption	39.4%	654
RF	RI	61.5%	130
RF	Absorption	58.8%	265

*k*NN: *k*-nearest neighbor; SVM: support vector machine; RF: random forest; RI: refractive index.

## Data Availability

Not applicable.
